# A Hematologic Twist: Zinc-Induced Copper Deficiency Mimicking Myelodysplastic Syndrome

**DOI:** 10.7759/cureus.87624

**Published:** 2025-07-09

**Authors:** Mary Hinson Mims, Erin S Reid, Mary Grace Hash, Andreas Maddux

**Affiliations:** 1 Internal Medicine, Edward Via College of Osteopathic Medicine, Auburn, USA; 2 Internal Medicine, Alabama College of Osteopathic Medicine, Dothan, USA; 3 Nephrology and Internal Medicine, University of Alabama at Birmingham (UAB) St. Vincent's East, Birmingham, USA

**Keywords:** denture adhesives, myelodysplastic syndromes, supplement risks, unexplained anemia, unexplained neutropenia, zinc excess, zinc-induced hypocupremia

## Abstract

Zinc-induced copper deficiency is an underdiagnosed condition that can lead to systemic manifestations of hypocupremia, including anemia, neutropenia, ataxic myelopathy, spastic paresis, alopecia, and skin depigmentation. This case illustrates the harmful effects of zinc supplementation and zinc-containing denture adhesive in a 68-year-old man, ultimately leading to copper deficiency. It also underscores the importance of considering zinc toxicity in the differential diagnosis of patients presenting with unexplained cytopenias, bone marrow abnormalities, and related symptoms. Furthermore, this report highlights the need for clinicians to consider zinc toxicity-induced copper deficiency in the evaluation of unexplained pancytopenias, especially in the post-COVID era, where zinc supplementation has become increasingly prevalent.

## Introduction

Zinc-induced copper deficiency is a clinically significant yet often underrecognized condition that can lead to a broad range of hematologic and neurologic abnormalities. While zinc is essential for immune function and enzymatic activity, excessive intake, particularly through oral supplementation and zinc-containing denture adhesives, can disrupt copper homeostasis [1,2]. Zinc induces the production of a protein in enterocytes that preferentially binds copper and prevents its absorption [3]. Since the COVID-19 pandemic, zinc supplementation has become increasingly prevalent, driven largely by heightened public awareness of immune health and a desire for preventative action against viral infections [4-7]. In Lebanon, for example, the proportion of adults reporting zinc supplement use rose significantly from 18.8% pre-pandemic to 29.3% during the pandemic [7]. As zinc supplementation for immune support becomes more widespread, the risk of trace mineral imbalances increases, particularly among vulnerable populations such as infants, pregnant and lactating women, adults over age 75, individuals taking medications like proton pump inhibitors, vegetarians and vegans with lower dietary zinc bioavailability, and those with occupational exposure to zinc fumes or dust [8,9].

Several studies have highlighted the link between excessive zinc exposure and copper deficiency [1,2]. Nations SP et al. reported cases of middle-aged denture-wearing patients with neurologic dysfunction and hypocupremia associated with chronic overuse of zinc-containing denture creams [2]. Similarly, Cathcart SJ and Sofronescu AG described a 36-year-old woman with a history of substance use who developed myeloneuropathy and hematologic abnormalities associated with prolonged use of zinc-containing denture adhesives [1]. Clinically, copper deficiency may present with sensory ataxia, myelopathy, anemia, neutropenia, alopecia, and skin depigmentation. Neurological deficits are particularly concerning, as they may be only partially reversible despite supplementation, emphasizing the importance of timely recognition and intervention [1,3,10]. These neurological features closely resemble subacute combined degeneration of the spinal cord, often leading to misdiagnosis and subsequent inappropriate treatment [1,2,10]. The potential for zinc-induced copper deficiency to cause significant hematologic and neurologic manifestations extends beyond its direct effects, as it can also closely resemble serious hematologic disorders, complicating diagnosis and management.

Copper deficiency can also mimic myelodysplastic syndrome (MDS) due to its impact on hematopoiesis. Copper is an essential cofactor for enzymes such as ceruloplasmin, which is involved in iron metabolism and the maturation of blood cells [11,12]. When copper is deficient, iron transport becomes disrupted, contributing to cytopenias such as anemia and neutropenia [11,12]. Additionally, bone marrow findings, including dysplastic myeloid precursors, vacuolization of erythroid and myeloid cells, and ringed sideroblasts, may closely resemble those of MDS [13]. This overlap can lead to misdiagnosis unless copper levels are specifically checked [13]. Unlike true MDS, however, copper deficiency is reversible with appropriate treatment, making early recognition critical for proper management [13].

In an era where supplement use has sharply increased, clinicians must maintain a broad differential when evaluating unexplained cytopenias [1,2]. This case highlights the importance of assessing trace mineral levels and considering reversible causes, such as zinc-induced copper deficiency, before diagnosing severe hematologic disorders like MDS, which require vastly different therapeutic approaches [11-13]. We present a case of zinc-induced copper deficiency that manifested with cytopenias and neurologic symptoms, initially raising concerns for MDS. This case underscores the importance of thoroughly evaluating trace mineral levels in patients with unexplained hematologic abnormalities.

## Case presentation

This case involves a 68-year-old male patient who visited the clinic for a follow-up consultation after his hospitalization, which was necessitated by a three-month history of progressive weakness, exertional dyspnea, and episodes of orthostatic dizziness. The patient’s medical history includes chronic kidney disease stage 3b, type 2 diabetes mellitus, essential hypertension, gout, and secondary hyperparathyroidism of renal origin. During his hospitalization, a complete blood count revealed severe anemia and neutropenia. A bone marrow biopsy was conducted, which showed hypocellular bone marrow (10-20%), a mildly increased blast population (up to 5%), and iron stores positive for ringed sideroblasts greater than 15%. Peripheral blood myeloid panel and MDS FISH were negative. He was discharged from the hospital with a presumed diagnosis of MDS, to be managed on an outpatient basis.

Upon further investigation, it was found that the patient had been using zinc-containing denture adhesives and taking zinc supplements for immune support. A complete blood count, iron levels, nutritional studies, and copper levels were rechecked. Laboratory findings, as detailed in Table 1, demonstrated pancytopenia with anemia, leukopenia, and low serum copper, in addition to abnormal iron studies and elevated ferritin.

**Table 1:** Laboratory values at hospital follow-up.

Component	Patient’s values	Normal range (Male)
WBC count	0.87 × 10³/µL	4.0-11.0 × 10³/µL
RBC count	2.75 × 10⁶/µL	4.5-5.9 × 10⁶/µL
Hemoglobin	8.6 g/dL	13.5-17.5 g/dL
Hematocrit	26.20%	41-53%
Mean corpuscular volume (MCV)	95.3 fL	80-100 fL
Mean corpuscular hemoglobin (MCH)	31.3 pg	27-33 pg
Mean corpuscular hemoglobin concentration (MCHC)	32.8 g/dL	32-36 g/dL
Red cell distribution width (RDW)	19.70%	11.5-14.5%
Absolute neutrophil count (ANC)	19.6%	40-70%
Platelet count	195 × 10⁹/L	150-450 × 10⁹/L
Vitamin B12	2000 pg/mL	200-900 pg/mL
Folate	14.56 ng/mL	3-20 ng/mL
Iron	27 µg/dL	60-170 µg/dL
Total iron-binding capacity (TIBC)	190 µg/dL	240-450 µg/dL
Ferritin	805 ng/mL	20-300 ng/mL
Transferrin saturation	14%	20-50%
Copper concentration	10 µg/dL	70-140 µg/dL

The patient was informed of the diagnosis; consequently, zinc supplementation and the zinc-containing denture adhesive were promptly withdrawn from his treatment regimen. He was also started on daily copper replacement therapy. The standard dosage for copper replacement therapy in cases of hypocupremia depends on multiple factors, including the severity of the deficiency and the route of administration. In our case, the patient was prescribed 2 mg/day of copper gluconate.

The patient's neutropenia, fatigue, weakness, and low copper levels resolved within three weeks of copper supplementation and discontinuation of zinc, as shown in the follow-up lab values in Table 2. He remains anemic, which may be attributed to his history of chronic kidney disease. Patient education regarding the potential adverse effects of supplementation was also addressed to help reduce the recurrence of these symptoms. The patient has been scheduled for a routine follow-up in three months to monitor laboratory values and assess for any persistent abnormalities.

**Table 2:** Follow-up labs after treatment.

Component	Patient’s values	Normal range (Male)
WBC count	4.03 × 10³/µL	4.0-11.0 × 10³/µL
RBC count	4.46 × 10⁶/µL	4.5-5.9 × 10⁶/µL
Hemoglobin	11.5 g/dL	13.5-17.5 g/dL
Hematocrit	36.80%	41-53%
Mean corpuscular volume (MCV)	82.5 fL	80-100 fL
Mean corpuscular hemoglobin (MCH)	25.8 pg	27-33 pg
Mean corpuscular hemoglobin concentration (MCHC)	31.3 g/dL	32-36 g/dL
Red cell distribution width (RDW)	16.10%	11.5-14.5%
Absolute neutrophil count (ANC)	71%	40-70%
Platelet count	257 × 10⁹/L	150-450 × 10⁹/L
Vitamin B12	2000 pg/mL	200-900 pg/mL
Folate	14.56 ng/mL	3-20 ng/mL
Iron	35 µg/dL	60-170 µg/dL
Total iron-binding capacity (TIBC)	233 µg/dL	240-450 µg/dL
Ferritin	713 ng/mL	20-300 ng/mL
Transferrin saturation	14.40%	20-50%
Copper concentration	76 µg/dL	70-140 µg/dL

## Discussion

The utilization of vitamins, supplements, and minerals has gained considerable prominence in society following the COVID-19 pandemic [4-7]. In this report, the patient had been taking daily zinc supplements to enhance immune health, in conjunction with a daily denture adhesive containing zinc, following a dental procedure for the past two years. The interaction between these two modalities resulted in significant anemia and neutropenia in the patient.

Zinc-induced copper deficiency is a relatively uncommon yet significant cause of anemia in individuals using zinc-containing denture adhesives or oral zinc supplementation [1-3]. Zinc is an essential mineral that, when absorbed in appropriate amounts, aids in protein and DNA synthesis and promotes healthy immune function [3]. However, many clinicians remain unaware of the biochemical pathway through which excess zinc intake can lead to copper deficiency, significantly contributing to the underdiagnosis of this condition [3,11,12]. When excess zinc is absorbed into the body, it increases enterocyte-mediated production of metallothionein [3,14].

**Figure 1:**
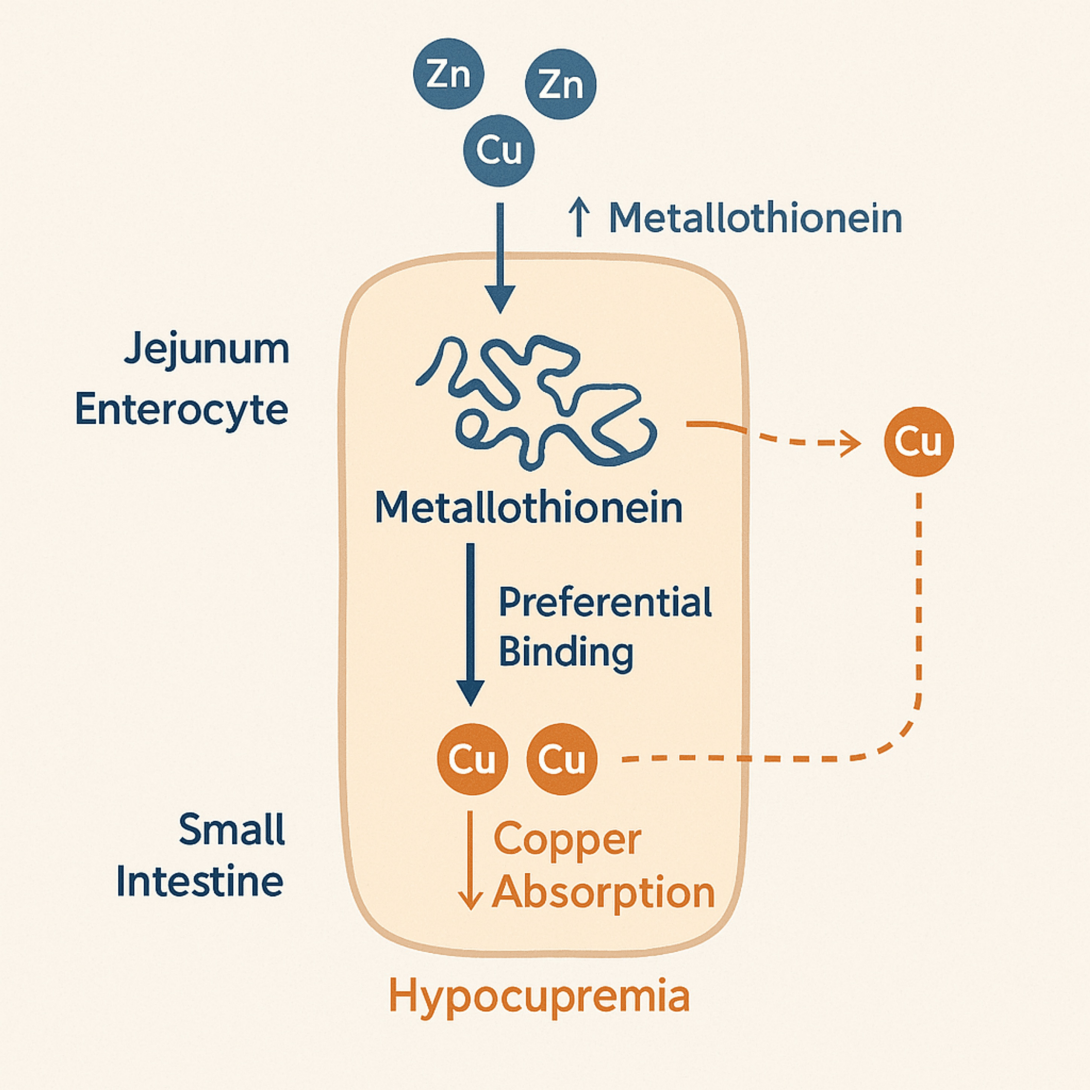
Mechanism of zinc-induced copper deficiency. This diagram illustrates how excess zinc disrupts copper absorption in the small intestine. In the jejunal enterocyte, elevated zinc levels stimulate increased production of metallothionein, a metal-binding protein. Metallothionein has a higher affinity for copper than for zinc, leading to preferential binding of copper ions. As enterocytes undergo natural turnover and are sloughed off, the copper bound to metallothionein is lost in the feces. This process reduces systemic copper absorption, ultimately resulting in hypocupremia. This mechanism explains the hematologic and neurologic manifestations observed in zinc-induced copper deficiency. This image was created using DALL·E, an AI-based illustration tool [15].

Metallothionein is a protein that protects the body from metal toxicity by binding metals like zinc and copper to neutralize their effects [3]. Increased zinc absorption signals the body to produce more metallothionein, helping to maintain zinc homeostasis [3]. However, metallothionein has a higher binding affinity for copper than for zinc [3,8,14]. As metallothionein levels rise, these proteins begin to preferentially bind to free copper in the jejunum [9]. The resulting metallothionein-copper complexes become trapped in the enterocytes and are ultimately shed into the feces as the enterocytes are sloughed off [3,14]. This cascade results in a significant decrease in copper absorption into systemic circulation, leading to hypocupremia. Figure 1 shows a visual summary of this mechanism [3,11,12].

Hypocupremia can manifest as a wide range of systemic symptoms, including anemia that is unresponsive to iron therapy, neutropenia, ataxic myelopathy, sensory ataxia, depigmentation, and cardiovascular effects [1]. The patient in our case exhibited findings significant for refractory anemia and neutropenia. Due to his anemic profile, he underwent a bone marrow biopsy, which revealed hypocellular bone marrow (10-20%), a mildly increased blast population (up to 5%), and iron stores positive for ringed sideroblasts greater than 15%. These bone marrow findings closely parallel those seen in MDS, often leading to misdiagnosis and inadequate treatment [16].

Ineffective bone marrow hematopoiesis has a broad differential diagnosis, including conditions such as MDS, which was initially considered in this case [17,18]. Like our patient, these syndromes can present with anemia, neutropenia, or thrombocytopenia. It is crucial to distinguish between anemia caused by zinc-induced hypocupremia and anemia resulting from MDS. Anemia due to secondary hypocupremia is reversible with the cessation of zinc supplementation and the initiation of copper replacement. Making this distinction can help prevent unnecessary and intensive interventions such as blood transfusions, allogeneic bone marrow transplantation, and erythropoiesis-stimulating agents, which are standard treatments for MDS [17,18].

In patients presenting with signs and symptoms of anemia, a thorough patient history is critical to identifying potential risks, such as zinc supplementation or the use of zinc-containing denture adhesives [1-3]. If clinical suspicion arises that excess zinc supplementation may be the cause, further investigation is warranted [1-3]. Key laboratory values to monitor include blood counts via CBC, iron studies, nutritional studies with zinc and copper levels, erythropoietin levels, and peripheral blood analyses such as fluorescence in situ hybridization (FISH), cytogenetics, and a myeloid panel [7,19]. In patients with excessive zinc supplementation, like our patient, laboratory results typically show a markedly elevated serum zinc concentration, a low or undetectable serum copper concentration, and may also indicate a low or undetectable level of ceruloplasmin [7].

## Conclusions

This case highlights the importance of considering zinc toxicity in the differential diagnosis of patients presenting with unexplained cytopenias, bone marrow abnormalities, and symptoms of anemia. The use of supplements has increased in recent years, particularly during the COVID-19 pandemic, underscoring the need for careful assessment in clinical practice. Early diagnosis and intervention, such as discontinuing excessive zinc intake and initiating copper supplementation, can help prevent misdiagnosis, avoid unnecessary treatments, and reduce the risk of long-term complications. Given the reversible nature of this condition, timely and appropriate management can lead to significantly improved patient outcomes, enhancing both health and quality of life.
